# Production of bacterial cellulose and enzyme from waste fiber sludge

**DOI:** 10.1186/1754-6834-6-25

**Published:** 2013-02-16

**Authors:** Adnan Cavka, Xiang Guo, Shui-Jia Tang, Sandra Winestrand, Leif J Jönsson, Feng Hong

**Affiliations:** 1 China-Sweden Associated Research Laboratory in Industrial Biotechnology, College of Chemistry, Chemical Engineering and Biotechnology, Donghua University, Shanghai, 201620, China; 2 Department of Chemistry, Umeå University, Umeå, SE-901 87, Sweden; 3 Group of Microbiological Engineering and Industrial Biotechnology, College of Chemistry, Chemical Engineering and Biotechnology, Donghua University, Shanghai, 201620, China

**Keywords:** Bacterial cellulose, *Gluconacetobacter xylinus*, Enzyme production, Cellulase, *Trichoderma reesei*, Fiber sludge

## Abstract

**Background:**

Bacterial cellulose (BC) is a highly crystalline and mechanically stable nanopolymer, which has excellent potential as a material in many novel applications, especially if it can be produced in large amounts from an inexpensive feedstock. Waste fiber sludge, a residue with little or no value, originates from pulp mills and lignocellulosic biorefineries. A high cellulose and low lignin content contributes to making the fiber sludge suitable for bioconversion, even without a thermochemical pretreatment step. In this study, the possibility to combine production of BC and hydrolytic enzymes from fiber sludge was investigated. The BC was characterized using field-emission scanning electron microscopy and X-ray diffraction analysis, and its mechanical properties were investigated.

**Results:**

Bacterial cellulose and enzymes were produced through sequential fermentations with the bacterium *Gluconacetobacter xylinus* and the filamentous fungus *Trichoderma reesei*. Fiber sludges from sulfate (SAFS) and sulfite (SIFS) processes were hydrolyzed enzymatically without prior thermochemical pretreatment and the resulting hydrolysates were used for BC production. The highest volumetric yields of BC from SAFS and SIFS were 11 and 10 g/L (DW), respectively. The BC yield on initial sugar in hydrolysate-based medium reached 0.3 g/g after seven days of cultivation. The tensile strength of wet BC from hydrolysate medium was about 0.04 MPa compared to about 0.03 MPa for BC from a glucose-based reference medium, while the crystallinity was slightly lower for BC from hydrolysate cultures. The spent hydrolysates were used for production of cellulase with *T. reesei*. The cellulase activity (CMCase activity) in spent SAFS and SIFS hydrolysates reached 5.2 U/mL (87 nkat/mL), which was similar to the activity level obtained in a reference medium containing equal amounts of reducing sugar.

**Conclusions:**

It was shown that waste fiber sludge is a suitable raw material for production of bacterial cellulose and enzymes through sequential fermentation. The concept studied offers efficient utilization of the various components in fiber sludge hydrolysates and affords a possibility to combine production of two high value-added products using residual streams from pulp mills and biorefineries. Cellulase produced in this manner could tentatively be used to hydrolyze fresh fiber sludge to obtain medium suitable for production of BC in the same biorefinery.

## Background

Production of high value-added materials from residual streams originating from renewable feedstock is an interesting possibility, which has the prospective of becoming an integral part of existing pulp mills and lignocellulosic biorefineries. One such material is bacterial cellulose (BC), which is a nano-structured material produced by various species of acetic acid bacteria [1]. BC is mainly built up by microfibrils, which are around 2–4 nm in diameter and which in turn build up fibers with an approximate size of less than 100 nm [2]. The fine and well-ordered structure of BC offers several advantages when it is used in matrices with other materials, such as low thermal expansion and superior reinforcement [3]. Already today, BC has reached a wide array of applications, such as health food, cosmetics, pharmaceutical and biomedical products, reinforcement of high-quality papers, diaphragms for electro-acoustic transducers, paint additives, coatings, reinforcement for optically transparent films, and proton-conducting membranes of fuel cells [1,3-7]. The future potential for BC is even wider than the already existing applications, especially if it can be produced in large amounts from an inexpensive feedstock, and may include areas such as specialty textiles [8], advanced functional materials, and packaging.

Production of bacterial cellulose from agricultural products and residues, which include konjak glucomannan [9], wheat straw [10,11], and cotton-based waste textiles [12], has previously been demonstrated. An advantage of using agricultural or industrial residual streams as feedstock for production of bacterial cellulose is the low cost of the raw material. When lignocellulosic feedstocks are pretreated at high temperature and high pressure they give rise to inhibitory compounds due to breakdown of polysaccharides and lignin. In the studies of Hong and Qiu [9] and Hong et al. [10], the hydrolysates obtained through acid hydrolysis of konjak glucomannan and wheat straw had to be detoxified using overliming in order to enable bacterial growth and production of BC.

Waste fiber sludge is a residual material originating from pulp mills and lignocellulosic biorefineries. Fiber sludge consists mainly of cellulose and hemicellulose, and usually has a low content of lignin (≤ 5%). Due to their composition and structure, fiber sludges are usually easy to be hydrolyzed enzymatically without prior thermochemical pretreatment, and could potentially yield hydrolysates with high glucose concentrations and low content of inhibitory compounds. A low content of inhibitory compounds should be advantageous for the bacterial strains used for production of BC. There are, however, drawbacks associated with enzymatic hydrolysis, especially the high cost for the hydrolytic enzymes used in the process.

The objectives of this study were to investigate the appropriateness of waste fiber sludge for production of BC, and the possibility to combine the production of BC with production of hydrolytic enzymes useful for degradation of lignocellulose. The fiber sludges used in this study were originated from a pulp mill using a sulfate-based process (kraft pulping) and from a lignocellulosic biorefinery using a sulfite-based process. In addition, we investigated the metabolic preferences of the bacterium, *Gluconacetobacter xylinus*, used for the production of bacterial cellulose and the filamentous fungus, *Trichoderma reesei* (*Hypocrea jecorina*), used for enzyme production by analyzing the consumption of different components in the culture medium. Sequential production of BC and enzyme would potentially give two high value-added products from a residual stream of very low or no value. Production of BC and enzyme could tentatively also be integrated with biofuel manufacture, which would benefit from production of high value-added co-products and from on-site production of hydrolytic enzymes.

## Results and discussion

Fiber sludges collected from mills operating sulfate- and sulfite-based processes were characterized chemically and subjected to enzymatic hydrolysis without any thermochemical pretreatment. The dry-matter content of the SAFS was 51.7%, while it was 43.7% for the SIFS. Compositional analysis (Table 1) indicates that SAFS consisted mainly of glucan (69.1%) and xylan (15.4%). SIFS consisted mainly of glucan (89.7%) and contained very low levels of other carbohydrates, such as mannan (2.7%) and xylan (1.7%). The content of lignin was low in both SAFS and SIFS (Table 1).

**Table 1:** Composition (% w/w) of sulfate (SAFS) and sulfite (SIFS) fiber sludges

**Fiber sludge**	**Arabinan**	**Galactan**	**Glucan**	**Mannan**	**Xylan**	**Lignin**	**Ash**
SAFS	0.3	0.2	69.1	3.3	15.4	3.5	3.6
SIFS	<0.02	0.1	89.7	2.7	1.6	0.8	1.7

**Figure 1:**
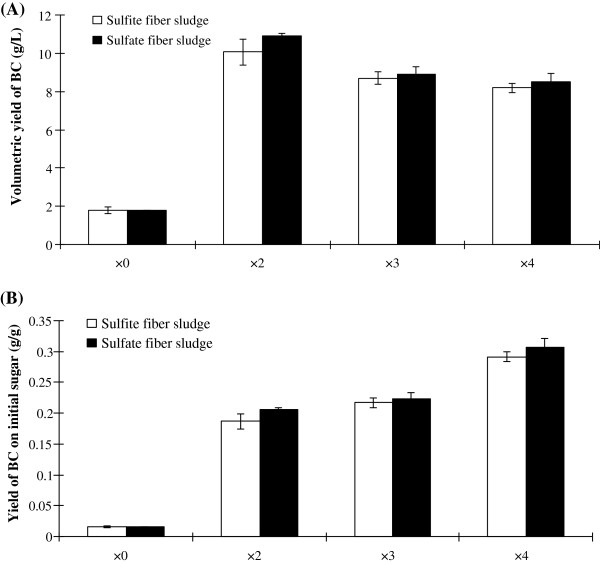
**Yields of bacterial cellulose (BC) after seven days of cultivation of *G. xylinus*.** The top graph **(A)** shows the volumetric yield [g BC (DW) per L] and the bottom graph **(B)** shows the yield on the initial amounts of reducing sugar [g BC (DW) per g initial sugar].

The first experimental series in which *G. xylinus* was grown on fiber sludge hydrolysates showed that it was advantageous to dilute the hydrolysates in order to facilitate BC production (Figure 1). The bacterium grew well in all diluted media. The results indicate that *G. xylinus* consumed similar amounts of reducing sugars in all of the experiments, namely around 20 g/L of reducing sugar during seven days of cultivation. The pH value of the media decreased during the cultivations, from around pH 5 to around pH 3.

The volumetric yield of BC (DW) in undiluted hydrolysate was about 2 g/L for both SAFS and SIFS. A two-fold dilution of the hydrolysate increased the volumetric BC yield to 11 g/L for SAFS and 10 g/L for SIFS, which represents a five-fold increase compared to undiluted hydrolysates (Figure 1A). When the hydrolysate was diluted three-fold, the volumetric yield of BC decreased slightly to around 9 g/L for both SAFS and SIFS. A four-fold dilution resulted in a BC yield of around 8 g/L (Figure 1A). The volumetric yield of BC (g/L) was not that different in the experiments with diluted hydrolysates. However, the BC yield on the initial amount of reducing sugar (g/g) increased with increasing dilution (Figure 1B). The two-fold dilution resulted in a BC yield of around 0.20 g/g, while the three-fold dilution resulted in an improvement to around 0.22 g/g. The four-fold dilution resulted in the highest yields, around 0.30 g of BC per g initial reducing sugar. The yield of BC on consumed reducing sugar was 0.5-0.6 g/g for the different dilutions of the SAFS hydrolysate. For the different dilutions of the SIFS hydrolysate, the yield of BC on consumed reducing sugar was about 0.4 g/g.

After evaluating the initial results, the four-fold dilution was selected for further experiments. The second attempt of production of BC was performed in larger scale and 100 mL of hydrolysate was used instead of 30 mL. Furthermore, a glucose reference with similar sugar content was included for comparison and the incubation time was increased to 14 days, after which the BC was collected through filtration and its properties were investigated in order to assess the outcome of the experiments. The results are summarized in Table 2. Surprisingly, the volumetric BC yield (DW) from the SAFS hydrolysate, 6.23 g/L, was higher than for the reference cultivation, which reached 4.90 g/L. The cultivation in SIFS hydrolysate gave 4.65 g/L, a slightly lower volumetric yield than for the reference. The water-holding capacity of the BC membranes was almost identical (Table 2). When the thickness of the strips was measured, the results were found to be different. The thickest BC strips, 3.11 mm, were found in the reference medium, which can be compared to 2.83 mm for the SAFS hydrolysate and 2.59 mm for the SIFS hydrolysate. The thickness of BC membranes is, however, less important than the tensile strength, since the thickness can be influenced by several factors, such as surface area and the amount of water held by the membrane. The BC membranes from SAFS and SIFS hydrolysates had a tensile strength of around 0.04 MPa (Table 2). This was considerably higher than for membranes produced in the reference medium, which exhibited resistance up to around 0.03 MPa. Similar results were found previously in a study of BC production from cotton-based waste textiles [12]. The fact that the tensile strength of BC produced in glucose-based medium was lower than that of BC produced in hydrolysates is perhaps due to the lower thickness of the BC pellicles from the hydrolysate media. The BC in the hydrolysate media would be able to form a more compact network than in the reference medium.

**Table 2:** Properties of BC produced in different media with a four-fold dilution of hydrolysate and after 14 days of fermentation

**Property/Culture medium**	**SAFS hydrolysate**	**SIFS hydrolysate**	**Reference medium**
Volumetric yield of BC (g/L)	6.23 ± 0.14	4.65 ± 0.15	4.90 ± 0.58
Water-holding capacity of BC (%)	99.5 ± 1.0	99.4 ± 2.0	99.5 ± 0.1
Thickness of BC strips (mm)	2.83 ± 0.20	2.59 ± 0.20	3.11 ± 0.22
Tensile force (N) (wet sheet)	0.48 ± 0.09	0.41 ± 0.08	0.39 ± 0.05
Tensile strength (MPa) (wet sheet)	0.042 ± 0.012	0.040 ± 0.020	0.031 ± 0.011
Degree of crystallinity (%)^[a]^	60.6	66.3	78.0

^[a]^ Calculations were based on the empirical method found in [26].

**Figure 2:**
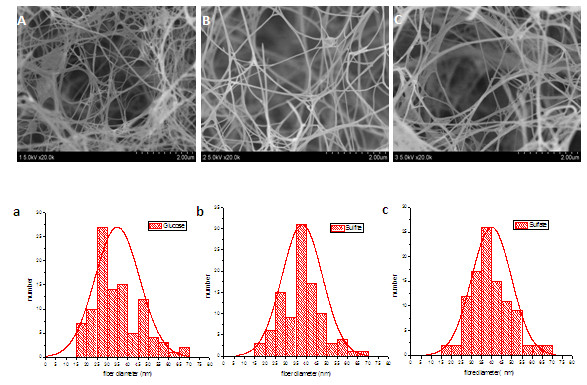
**SEM of freeze-dried BC from cultures with (A) glucose-based reference medium, (B) SIFS hydrolysate, and (C) SAFS hydrolysate.** The BC was harvested after 14 days. Figures **a**, **b**, and **c** show results from calculations of the diameter of fibers from glucose-based reference medium, SIFS hydrolysate, and SAFS hydrolysate, respectively.

The degree of crystallinity of the BC membranes differed depending on the cultivation medium. A higher degree of crystallinity gives rise to a more stable cellulose polymer when it comes to resistance to digestion and breakdown of the polymer. Hence, more crystalline cellulose polymers are also more difficult to solubilize and add functional groups to, which would be required if the cellulose is to be used for many of the potential applications for BC. The analysis of the different membranes showed that the most crystalline cellulose was found in BC from cultures with reference medium, where the degree of crystallinity was around 78%. The cellulose from SAFS hydrolysate had a crystallinity of around 61%, while the cellulose from SIFS hydrolysate had a crystallinity of around 66%. These results are further supported by scanning electron microscope (SEM) images, which display the structure of the cellulose membranes at a magnification of 20,000 times. Field-emission scanning electron micrographs (FESEM) of freeze-dried BC pellicles prepared from reference medium and from SIFS and SAFS hydrolysates are shown in Figure 2A-C. The fracture surface morphology of the BC pellicles from different media is essentially the same and the histograms (Figure 2a-c) based on the FESEM images illustrate a small average diameter and a relatively narrow diameter distribution for the nanofibers of the BC pellicles. The fiber distribution data indicate that most of the fibers were in the range 15 to 70 nm, with an average width of 35 to 40 nm.

In the experiment in which the cultures were harvested after 14 days, the yield of BC on consumed reducing sugar was 0.50 g/g for the SAFS hydrolysate and 0.31 g/g for the SIFS hydrolysate. The BC yield from the cultures with reference medium was only 0.28 g/g. The yield of BC on the initial concentration of reducing sugar was 0.22 g/g for the SAFS hydrolysate, 0.17 g/g for the SIFS hydrolysate, and 0.18 g/g for the reference medium.

The results obtained in our study compare well to results achieved in other studies where various substrates have been used for production of BC. Keshk and Sameshima [13] studied the ability of *G. xylinus* ATCC10245 to produce BC using sixteen different carbon sources including monosaccharides, polysaccharides, and alcohols. Only four of these carbon sources turned out to be useful for production of BC. The highest BC yield on the consumed amount of carbon source, 0.287 g/g, was achieved with glycerol [13]. Fructose, glucose, and inositol gave BC yields amounting to 0.153 g/g, 0.087 g/g, and 0.078 g/g, respectively. The high yield achieved with glycerol could be attributed to the low consumption rate for this carbon source. Thompson and Hamilton [14] compared two different *G. xylinus* strains, ATCC10821 and ATCC23770, and their ability to grow on various carbon sources, such as potato effluents, cheese whey permeate, and sugar beet raffinate. Their results indicated that the highest yield for ATCC10821 was achieved after seven days of incubation, while ATCC23770 gave better yield after 14 days. The best results were achieved with potato effluents (starch) and ATCC23770, which gave a BC yield on consumed glucose of 0.27 g/g after 14 days, nearly the same yield as that obtained from a cultivation on optimized glucose medium [14]. This can be compared to our study, where the yields on consumed reducing sugars for the cultivations on SAFS (0.50 g/g) and SIFS (0.31 g/g) hydrolysates were higher than that of the cultivation on glucose-based reference medium (0.28 g/g). This suggests that BC production from residual low-value streams from forest biorefineries performs well compared to BC production from residues originating from the agricultural sector.

The analysis of monosaccharides is summarized in Table 3. The results indicate that glucose was the main nutrient source and that it was consumed efficiently in all cultivations. The analysis also suggests that some of the xylose was consumed. The utilization of xylose by *G. xylinus* is not well understood but it is suggested in the literature that most *G. xylinus* strains have poor ability to utilize xylose [15,16]. The metabolic pathway presented by Ross et al. [17] suggests that *G. xylinus* has the ability to utilize xylose. It has been proposed that xylose is mainly used as a source of energy for the bacterium, as it is consumed by *G. xylinus* through conversion into glyceraldehyde-3-phosphate, which in turn ends up in the tricarboxylic acid cycle (TCA) as acetyl Co-A after several other reactions [17]. Our study of complex fermentation media composed of fiber sludge hydrolysates further supports the suggestion that *G. xylinus* does convert xylose. The analysis of the media before and after fermentation with *G. xylinus* indicates that the consumption of xylose was around 2 g/L during 14 days of fermentation (Table 3).

**Table 3:** **Analysis of media used for cultivation of*G. xylinus***

**A. Prior to fermentation with * G. xylinus*and with a four-fold dilution of the hydrolysates.**						
**Media/Composition**	**Glucose (g/L)**	**Xylose (g/L)**	**Arabinose (g/L)**	**Galactose (g/L)**	**Mannose (g/L)**	**Acetic acid (g/L)**
SAFS hydrolysate	14.1 ± 0.3	3.3 ± 0.1	< 0.1	< 0.1	< 0.1	< 0.1
SIFS hydrolysate	17.6 ± 0.2	0.2 ± 0.1	< 0.1	< 0.1	< 0.1	< 0.1
Reference medium	18.3 ± 0.3	-	-	-	-	-
**B. After 14 days fermentation with *G. xylinus *(with a four-fold dilution of the hydrolysates).**						
**Media/Composition**	**Glucose (g/L)**	**Xylose (g/L)**	**Arabinose (g/L)**	**Galactose (g/L)**	**Mannose (g/L)**	**Acetic acid (g/L)**
SAFS hydrolysate	< 0.1	1.8 ± 0.1	< 0.1	< 0.1	< 0.1	< 0.1
SIFS hydrolysate	< 0.1	0.2 ± 0.1	< 0.1	< 0.1	< 0.1	< 0.1
Reference medium	0.6 ± 0.1	-	-	-	-	-

**Figure 3:**
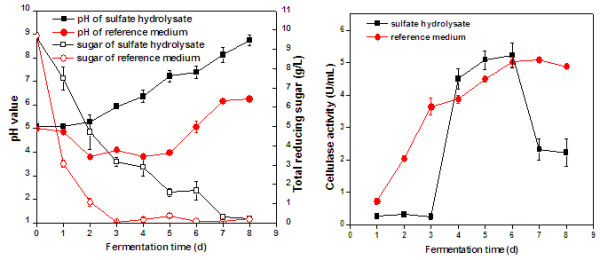
**Cultivations of *T. reesei *in SAFS spent hydrolysate and reference medium supplemented with 2%waste fiber sludge (SAFS).** The figure shows the concentration of reducing sugar and the pH value (left) and the cellulase (CMCase) activity (right).

**Figure 4:**
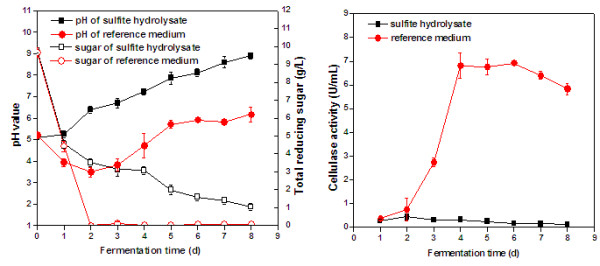
**Cultivations of*T. reesei*in SIFS spent hydrolysate and reference medium supplemented with 2%waste fiber sludge (SIFS).** The figure shows the concentration of reducing sugar and the pH value (left) and the cellulase (CMCase) activity (right).

The concept of sequential fermentation has previously been successful for production of ethanol and enzymes from lignocellulosic feedstock [18] and waste fiber sludge [19]. This concept offers several potential advantages. These include efficient utilization of the various components in the cultivation medium by two different microorganisms. In the present study, the possibility to produce two value-added products, BC and enzymes, was also investigated, and *T. reesei* was used in the second step rather than *Aspergillus niger (A. niger)*, which was used in previous studies [18,19]. With the production of enzymes in the second fermentation step, the need of external supply of enzymes for hydrolysis of fiber sludge would decrease, as enzymes could tentatively be supplied through on-site enzyme production. The results achieved in this study indicate that the spent SAFS hydrolysate served as a good medium for enzyme production with *T. reesei*. The cellulase activity after 6 days of fermentation reached 5.2 U/mL (87 nkat/mL) (Figure 3). *T. reesei* seemed to grow well in the medium despite its low content of easily accessible monosaccharides that the fungus could use as carbon source (cf. Table 3). However, in addition to monosaccharides and acetic acid (Table 3), the medium may also contain disaccharides, oligosaccharides, and other substances that the fungus could utilize as carbon source. The glucose-based reference medium and the spent SAFS hydrolysate supplemented with fresh sulfate fiber sludge produced almost equal levels of cellulase activity (Figure 3). Cultivations in glucose-based reference medium supplemented with sulfite fiber sludge did exhibit higher activity (Figure 4) than the cultivations in reference medium with sulfate fiber sludge or spent SAFS hydrolysate medium (Figure 3), but the cultivations in spent SIFS did not result in any substantial enzyme activity (Figure 4). These results were somewhat surprising, since it is not clear that the media contained compounds that inhibit *T. reesei*. Analysis of the raw material showed that none of the fiber sludges contained any detectable concentrations of sulfite [<5 mg/kg (DW)]. Both fiber sludges contained some sulfate, namely 100 mg/kg (DW) for the SAFS and 200 mg/kg for the SIFS. As only very small amounts (2% w/v) of the sludges were added as supplement to the cultivation media used for enzyme production, the sulfate content is unlikely to have had any negative effect on the microorganism. Since reducing sugar was consumed and the pH changed (Figure 4), the problem may instead be related to cellulase production.

**Figure 5:**
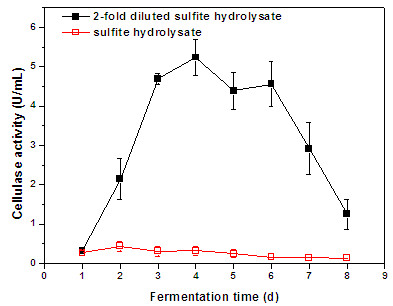
**Cellulase (CMCase) activity in cultivations of *T. reesei *in SIFS spent hydrolysate with and without dilution of the medium.**

An attempt was made to improve enzyme production by *T. reesei* by dilution of the spent SIFS medium. With a two-fold dilution with water, addition of 2% (w/v) SIFS, and adjustment of the level of reducing sugars to 10 g/L using glucose, *T. reesei* produced a cellulase activity level of 5.2 U/mL (Figure 5) after 4 days of cultivation, the same level of activity that was obtained using spent SAFS medium. More research is needed to elucidate why undiluted spent SIFS medium inhibited or failed to induce enzyme production.

Several other studies have been performed with *T. reesei* Rut C-30 and complex carbon sources derived from lignocellulose. Shin et al. [20] investigated several different cellulose-based media. In one of these experiments, a mixture of milled newspaper (2%) and lactose (1%) was used as substrate for enzyme production and the highest activity reached was 30 nkat/mL (1.8 FPU/mL). The same study reported about experiments performed with fiber residues from hydrolyzed office paper (2%), in which the highest enzymatic activity was 45 nkat/mL (2.7 FPU/mL). Furthermore, pretreated newspaper (1%) and pretreated oak wood (2%) were also used for enzyme production in the same study. In experiments with newspaper and oak media, the cellulase activity reached levels of 25 nkat/mL (1.5 FPU/mL) and ~72 nkat/mL (4.3 FPU/mL), respectively. Pretreated wood has been used for enzyme production with *T. reesei* Rut C-30 in other studies as well. Szengyel et al. [21] used steam-pretreated willow as substrate and achieved 30 nkat/mL (1.8 FPU/mL) as the highest activity. This was obtained in experiments where 50% fibrous pretreated willow was mixed with 50% of concentrated filtrate (liquid). The medium contained around 20 g/L of carbohydrate [21]. Softwood has also been used for enzyme production in a similar manner. Szengyel et al. [22] used steam-pretreated spruce as the carbon source. Several different experiments were performed and 2% washed steam-pretreated spruce gave the highest activity, namely ~13 nkat/mL (0.8 FPU/mL). Although it is evident that a variety of cellulosic media can be used for enzyme production with *T. reesei*, our study differs from the others by the utilization of a waste stream (spent hydrolysate) and by that the enzyme production step was preceded by a BC production step, which makes the enzymes a secondary product.

## Conclusions

In conclusion the results of this study show that there is a great potential in utilizing waste fiber sludge for co-production of bacterial cellulose and enzymes. Fiber sludge serves as a low cost and abundant raw material which is easily hydrolyzed to sugars without pretreatment. The results also indicate that production of BC from fiber sludge hydrolysates gives a cellulose polymer displaying superior properties compared to the one produced from a glucose-based reference medium. Conditioning of hydrolysates and optimization of the cultivation conditions are likely to result in higher volumetric yields than what reported here and deserve attention in future studies.

## Methods

### Fiber sludges

The waste fiber sludges that were used in this study were kindly provided by two different European mills, one pulp and paper mill using a sulfate-based process (SAFS) and a lignocellulosic biorefinery using a sulfite-based process (SIFS). The characterization and analysis of the feedstocks and the content of monosaccharides, lignin and ash was performed by MoRe Research (Örnsköldsvik, Sweden).

### Enzymatic hydrolysis

The hydrolysis of the fiber sludges was performed enzymatically without any prior thermochemical pretreatment. Initially, 290 g of moist SAFS with a dry-weight content of 51.7% were mixed with 693.8 g of citrate buffer (0.05 M, pH 5.0), while 343 g of moist SIFS with a dry-weight content of 43.7% were mixed with 640.8 g of the citrate buffer. The fiber sludges were mixed in 2-L shake flasks, the final dry-matter content was 15% (w/w), and the total content per shake flask was 1 kg. The enzyme preparation used for hydrolysis was Cellic CTec2 (Novozymes, Bagsvaerd, Denmark). The enzyme preparation was added to a final concentration of 1.6% (w/w) of the reaction mixture, which corresponded to 10 FPU/g biomass (dry weight of waste fiber sludge). The flasks were incubated with orbital shaking (Ecotron, Infors AG, Bottmingen, Switzerland) at 50°C and 150 revolutions per minute (rpm) for 48 h. The glucose level during hydrolysis was monitored using a glucometer (Glucometer Elite XL, Bayer Healthcare, Leverkusen, Germany). After hydrolysis, the slurries were centrifuged (Allegra X-22R, Beckman Coulter, Brea, CA, USA) at 4°C and 4,200 *g* for 10 min to recover the liquid fractions. The pH of the liquid fractions was adjusted to 2.0 with sulfuric acid, and they were then stored in a freezer before further processing.

### Production of bacterial cellulose

Production of BC was performed using *Gluconacetobacter xylinus* (*Acetobacter xylinus*) ATCC 23770 (American Type Culture Collection, Manassas, VA, USA). A series of 100-mL flasks were filled with 30 mL fiber sludge hydrolysate and supplemented with 5 g/L yeast extract and 3 g/L tryptone. Fiber sludge hydrolysates were either undiluted or diluted two-fold, three-fold and four-fold. The flasks were autoclaved at 105°C for 30 min in order to sterilize the growth media. The flasks were inoculated with 10% (v/v) *G. xylinus* inoculum, which was pre-grown for 24 h in a synthetic medium (25 g/L D-glucose, 5 g/L yeast extract and 3 g/L tryptone, pH 5.0). The flasks were incubated statically at 30°C for 7 days, after which the yield of BC and the pH value were measured. Samples of the culture fluid were taken for analysis of the monosaccharide content.

A second series of experiments consisted of four-fold diluted fiber sludge hydrolysates and a glucose reference with similar monosaccharide content. The experiments were performed as described above but with 100 mL of medium in 250-mL flasks. The time of incubation was increased from 7 days in the first experiment to 14 days. After 14 days of static incubation, the BC membranes were collected by filtration and were then dried to constant weight at 105°C. After that, the BC was weighed for calculation of the yield.

The yield of BC on initial reducing sugar (g/g) was calculated by dividing the volumetric yield of BC with the initial concentration of reducing sugar. The yield of BC on consumed sugar (g/g) was calculated by using the following equation:

$$\mathrm{BC}\;\mathrm{yield}\;\mathrm{on}\;\mathrm{consumed}\;\mathrm{sugar}\;\left(g/g\right)\;=\;\frac{\mathrm{BC}\left(g\right)}{\mathrm{Initial}\;\mathrm{reducing}\;\mathrm{sugar}\;-\;\mathrm{residual}\;\mathrm{sugar}\;\left(g\right)}$$

For characterization of BC membranes, the cellulose pellicle was soaked in a 0.1 M solution of sodium hydroxide (60 min, 80°C) to remove impurities, such as culture medium and trapped bacterial cells. A second wash was performed with deionized water at the same temperature and for the same period of time. The BC pellicle was then washed with deionized water until the pH of washing water was neutral.

### Production of cellulase

Enzyme production with *Trichoderma reesei* Rut C-30 was performed by using spent hydrolysates obtained after BC production, and with the addition of 2% (w/v) of the corresponding dried waste fiber sludge. Reference medium, which was used as a control, was based on glucose (10 g/L) with 2% (w/v) additional waste fiber sludge. Separate cultures with reference medium were used for SAFS and SIFS. Each of a series of 500-mL flasks contained 100 mL spent hydrolysate supplemented with 0.1% (w/v) tryptone, 0.05% citric acid, 2% Vogel’s media [23], and 0.015% Tween 80. The flasks containing the media were autoclaved at 110°C for 30 min. The flasks were then inoculated with 10% (v/v) of a suspension of *T. reesei* pellets from a culture with glucose-based reference medium that was pre-grown at 30°C for 36 h. The cultivations were carried out at 28°C and 160 rpm for the following 8 days.

### Enzyme activity assay

The cellulase activity was measured using a reducing sugar assay developed for determination of xylanase activity and based on dinitrosalicylic acid (DNS) [24], but with 1% (w/v) of carboxymethyl cellulose (CMC) as substrate instead of xylan, and using a buffer consisting of 50 mM citric acid (pH 5.0). A mixture containing 0.9 mL substrate solution and 0.1 mL enzyme sample was incubated at 50°C for 10 min. Blanks containing (A) enzyme but no substrate and (B) substrate but no enzyme were included with all assays of enzyme activity, and the values obtained for the samples were corrected using the blank values. One unit of cellulase activity equals formation of 1 μmol glucose from CMC per min at pH 5.0 and 50°C. The activity of the culture fluids were calculated as the volumetric activity (U/mL) [one unit (U) equals 16.67 nkat].

### Analysis of sugars and acetic acid

The concentrations of arabinose, galactose, glucose, xylose, and mannose were determined using high-performance anion-exchange chromatography (HPAEC). The system used was an ICS-3000 from Dionex (Sunnyvale, CA, USA) with an electrochemical detector. The separation was performed with a CarboPac PA20 (3 × 150 mm) separation column equipped with a CarboPac PA20 (3 × 30 mm) guard column (Dionex). Elution was performed with a 2 mM solution of NaOH during 25 min, followed by regeneration at 5 min with 100 mM NaOH, and equilibration for 15 min with 2 mM NaOH (Sodium hydroxide solution for IC, Sigma-Aldrich, Steinheim, Germany). The flow rate was 0.4 mL/min.

The concentration of acetic acid was determined with HPAEC by using the ICS-3000 system and its conductivity detector. The separation was performed with an IonPac AS15 (4 × 250 mm) separation column equipped with an IonPac AG15 (4 × 50 mm) guard column (Dionex). The mobile phase consisted of a 35 mM solution of NaOH (Sodium hydroxide solution for IC, Sigma-Aldrich), and the flow rate was 1.2 mL/min.

### Tensile strength of wet BC membranes

The washed BC pellicle was cut into 40 mm long and 10 mm wide strips for analysis of tensile strength. The tensile strength of the wet BC was measured by using a universal testing machine (H5K-S, Hounsfield Test Equipment Ltd., UK) operating at a crosshead speed of 50 mm/min. All data for determination of tensile strength were collected under the same conditions. The tensile strength (in megapascal, MPa, or N/mm^2^) was calculated by dividing the tensile force by the area of the cross section of the BC strips. Each test was performed by using 10 samples and mean values of the strength of BC are given.

### Electron microscopy

The purified BC pellicle was dried by using a vacuum freeze dryer (LyoQuest-55 Plus, Telstar, Spain) and the samples were then coated with gold (E-1045, Hitachi, Tokyo, Japan). Analysis of the structure of the BC was performed by using a Field-Emission Scanning Electron Microscope (FESEM) (S-4800, Hitachi) at 15, 25 or 35 kV. The amplification was 20,000. The analysis of the diameter of 100 fibers was performed by using the software ImageJ [25].

### Degree of crystallinity of BC

X-ray diffraction (XRD) was used to examine the crystallinity of freeze-dried BC after alkaline washing. XRD spectra were recorded by using a D/Max-2550PC diffractometer (Rigaku, Tokyo, Japan) at 40 kV and 200 mA. Angular scanning was performed at 5-60° (2θ) and 1°/min. Calculations of the degree of crystallinity were based on the empirical method of Segal et al. [26].

## Abbreviations

BC: Bacterial cellulose; SAFS: Fiber sludge from sulfate process; SIFS: Fiber sludge from sulfite process; G. xylinus: *Gluconacetobacter xylinus*; T. reesei: *Trichoderma reesei*; DW: Dry weight; CMC: Carboxymethyl cellulose; SEM: Scanning electron microscope; FESEM: Field-emission scanning electron micrographs; TCA: Tricarboxylic acid cycle; A. niger: *Aspergillus niger*; rpm: Revolutions per minute; DNS: Dinitrosalicylic acid; HPAEC: High-performance anion-exchange chromatography; XRD: X-ray diffraction

## Competing interests

The authors declare that they have no competing interests.

## Authors’ information

AC and XG are doctoral students with interests in the areas of enzymatic saccharification and bioconversion of biomass for the production of value-added products including biofuels and biopolymers. SJT was a master student and now has a job in the biomedical area. SW is a postdoctoral researcher with interest in enzyme chemistry and technology. LJJ is a professor of biochemistry and biotechnology. His research is focused on biotechnology for the biorefining of lignocellulose. He is leader of the Biochemical Platform of the Bio4Energy research initiative (http://www.bio4energy.se). FH is a professor of biotechnology and bioengineering. His interests include low-cost production of bacterial cellulose and enzymes, bioconversion of renewable resources to high value-added products, as well as applications of biomaterials in biomedicine and functional materials.
